# *mTOR* eosinophilic renal cell carcinoma: a distinctive tumor characterized by mTOR mutation, loss of chromosome 1, cathepsin-K expression, and response to target therapy

**DOI:** 10.1007/s00428-023-03688-2

**Published:** 2023-11-08

**Authors:** Anna Caliò, Stefano Marletta, Giulio Settanni, Mimma Rizzo, Stefano Gobbo, Serena Pedron, Lavinia Stefanizzi, Enrico Munari, Matteo Brunelli, Lisa Marcolini, Anna Pesci, Stefano Fratoni, Francesco Pierconti, Maria Rosaria Raspollini, Antonio Marchetti, Claudio Doglioni, Mahul B. Amin, Camillo Porta, Guido Martignoni

**Affiliations:** 1 Department of Diagnostic and Public Health, Section of Pathology, University of Verona, Largo L. Scuro 10, 37134 Verona, Italy; 2 Department of Pathology, Pederzoli Hospital, Peschiera del Garda, Italy; 3 Department of Pathology, IRCCS Sacro Cuore Don Calabria Hospital, Negrar, Italy; 4 Division of Medical Oncology, A.O.U. Consorziale Policlinico Di Bari, Bari, Italy; 5 Department of Translational Medicine, University of Ferrara, Ferrara, Italy; 6 Department of Molecular and Translational Medicine, University of Brescia, Brescia, Italy; 7 Division of Anatomic Pathology, S. Eugenio Hospital, Rome, Italy; 8 Division of Anatomic Pathology and Histology, Foundation “A. Gemelli” University Hospital, Università Cattolica del Sacro Cuore, Rome, Italy; 9 Histopathology and Molecular Diagnostics, Azienda Ospedaliero Universitaria Careggi, Florence, Italy; 10 Division of Anatomic Pathology and Histology, Ospedale Clinicizzato “SS. Annunziata” Università Di Chieti, Chieti, Italy; 11 Department of Pathology, San Raffaele Hospital, Milan, Italy; 12 Department of Pathology and Laboratory Medicine, University of Tennessee Health Science, Memphis, TN USA; 13 Department of Urology, USC Keck School of Medicine, Los Angeles, CA USA; 14 Interdisciplinary Department of Medicine, University of Bari “A. Moro, Bari, Italy

**Keywords:** Cathepsin-K, Eosinophilic-RCC, *mTOR*, mTOR inhibitors, Next-generation sequencing, Oncocytic, RCC

## Abstract

In the spectrum of oncocytic renal neoplasms, a subset of tumors with high-grade-appearing histologic features harboring pathogenic mutations in mammalian target of rapamycin (mTOR) and hitherto clinical indolent behavior has been described. Three cases (2F,1 M) with histologically documented metastases (lymph node, skull, and liver) were retrieved and extensively investigated by immunohistochemistry, FISH, and next-generation sequencing. Tumors were composed of eosinophilic cells with prominent nucleoli (G3 by ISUP/WHO) arranged in solid to nested architecture. Additionally, there were larger cells with perinuclear cytoplasmic shrinkage and sparse basophilic Nissl-like granules, superficially resembling the so-called spider cells of cardiac rhabdomyomas. The renal tumors, including the skull and liver metastases, showed immunoexpression PAX8, CK8-18, and cathepsin-K, and negativity for vimentin. NGS identified *mTOR* genetic alterations in the three cases, including the skull and liver metastases. One patient was then treated with Everolimus (mTOR inhibitors) with clinical response (metastatic tumor shrinkage). We present a distinct renal tumor characterized by high-grade eosinophilic cells, cathepsin-K immunohistochemical expression, and harboring *mTOR* gene mutations demonstrating a malignant potential and showing responsiveness to mTOR inhibitors.

**Supplementary Information:**

The online version contains supplementary material available at 10.1007/s00428-023-03688-2.

## Introduction

During the last decade, our understanding of renal cell carcinoma (RCC) has vastly improved due to careful morphological evaluation of cases with correlation of data from high-throughput molecular profiling. This is particularly notable in oncocytic tumors where analysis of cases that were not easily classifiable as typical oncocytoma and chromophobe RCC has led to the establishment of additional clinicopathologically RCCs or a broadening of the spectrum within previously known RCC subtypes [1] including eosinophilic solid and cystic (ESC)-RCC [2, 3], succinate dehydrogenase (SDH)-deficient RCC [4], low-grade fumarate hydratase (FH)-deficient RCC [5], MiTF family translocation RCC [6], and TFEB-amplified RCC [7, 8].

Furthermore, increasing data regarding RCCs harboring mammalian target of rapamycin (mTOR) and tuberous sclerosis complex (TSC) mutations have been published. In detail, it has been known that alterations of the mTOR pathway are implicated in the pathogenesis of renal tumors arising in patients affected by the inherited tuberous sclerosis syndrome such as renal angiomyolipoma and related lesions [9, 10] and TSC-RCC [11, 12]. On the other hand, mutations of *TSC1/TSC2* and *mTOR* genes have also been found in some sporadic renal cell neoplasms including RCC with leiomyomatous stroma [13], ESC-RCC [14–16], chromophobe RCC [17], epithelioid angiomyolipoma/pure epithelioid PEComa [10], low-grade oncocytic tumor (LOT) [18, 19], and eosinophilic vacuolated tumor (EVT), among others [20, 21]. Although an indolent clinical course has been accustomed to most of such tumors, recently, Tjota et al. reported the first case of an eosinophilic tumor harboring *mTOR* gene mutation with liver metastasis [22]. In this study, we describe the clinical, morphological, immunohistochemical, and molecular characteristics of three additional cases of eosinophilic RCC harboring *mTOR* gene mutations with histologically documented metastases and, in one case, the clinical response to targeted therapy.

## Methods

### Patients and samples

From our archives of unclassified oncocytic renal tumors, we identified four previously unreported high-grade eosinophilic renal cell tumors from three different patients. All of them were consult cases. All procedures performed in our study involving human participants received approval (Prog. 4136CESC) and were in accordance with the ethical standards of the institutional and/or national research committee and with the declaration of Helsinki. All patients gave their written informed consent to diagnostic procedures and treatment according to institutional rules for everyday clinical practice and experimental evaluations on archival tissue. All slides (28 slides for case 1, 14 slides for case 2, 9 slides for case 3) were reviewed by an experienced pathologist (GM). Samples of both primary tumor and metastases were available.

### Immunohistochemistry

Sections from tissue blocks of primary and metastatic samples were immunohistochemically stained with the antibodies listed in Supplementary Table 1. All samples were processed using a sensitive Bond Polymer Refine detection system in an automated Bond immunohistochemistry instrument (Leica-Biosystems, Germany). The appropriate positive and negative controls were concurrently carried out. Labeling for each marker was recorded as the percentage of positive cells. P70S6 Kinase and ph4E-BP1 were performed in all samples using an automated Ventana Discovery system (Roche).

### Fluorescence in situ hybridization (FISH)

FISH was carried out on primary and metastatic samples using dual-color break-apart TFE3 and TFEB probes (Cytotest, USA) and 1p36/1q25 probe, spectrum-orange/spectrum-green (Vysis) as previously described [23]. Scoring was performed by two experienced pathologists (AC and MB). At least 100 neoplastic non-overlapping nuclei were included in the scoring. To avoid false positive results due to nuclear truncation, cells with a single fluorescent signal were not evaluated.

### Next-generation sequencing

#### DNA extraction

Sections were cut from all FFPE tissue blocks of primary and metastatic samples and manually microdissected to isolate a high percentage of neoplastic cells (> 50%). DNA was isolated using the GeneRead DNA FFPE kit (Qiagen, Hilden, Germany, http://www.qiagen.com Cat. n. 180,134). DNA quality and amount were assessed using NanoDrop and Qubit instruments (Thermo-Fisher Scientific) following the manufacturer’s instructions.

#### Library preparation and deep amplicon sequencing

We performed deep sequencing of the whole coding region and intron–exon junctions of 17 kidney-cancer-related genes with a custom panel created using the Ampliseq Designer pipeline (Thermo-Fisher Scientific) as previously described [3]. The genes included: *TSC1*, *TSC2*, *MTOR*, *AKT1*, *PIK3CA*, *PTEN*, *SDHB*, *FH*, *VHL*, *SETD2*, *BAP1*, *PBRM1*, *MET*, *FLCN*, *SMARCA4*, *SMARCB1*, and *TCEB1*.

#### Variant calling

Data from the PGM sequencing were initially processed using the Ion Torrent platform-specific software (Torrent Suite AD 5.6.4) to generate sequence reads, alignment of the reads on the reference genome Hg19, trim adapter sequences, filter, and remove poor signal-profile reads. The variant calling from the sequencing data was generated using the Variant Caller plugin.

To provide reliable somatic variant analysis we considered suitable only samples with more than 400,000 reads, an average coverage >  × 500, and a coverage uniformity > 95%. We applied the following filters to the Variant Caller plugin: minimum allele frequency value of 2% and minimum phred quality score of 30. Variant annotation and copy number variation analysis were performed using the Ion Reporter 5.12 software (Thermo-Fisher Scientific).

Variant annotations were also assessed using the Ensembl Variant Effect Predictor pipeline of the Wellcome Trust Sanger Institute [24] as a second database check. Filtered variants were visually examined using the Integrative Genomic Viewer tool to taste their level of quality and to confirm the variant presence on both “ + ” and “ − ” strands. The clinical relevance (pathogenicity) of the annotated variants was assessed using the COSMIC database (Wellcome Sanger Institute), OncoKB database [25], ClinVar (NCBI), and LOVD (IARC).

## Results

### Clinical and pathological features

The clinical and pathological features are summarized in Table 1. Two patients were female and one male. The patients’ ages at diagnosis were 21, 58, and 49 years respectively. None of the three patients showed clinical stigmata of tuberous sclerosis. Two of them underwent radical nephrectomy and one partial nephrectomy; one patient had two tumors simultaneously affecting the left kidney. The tumors ranged in size from 3.1 to 9.5 cm, and were all solid and brownish in color. The original diagnosis made at the referral institution was unclassified RCC in case 1, oncocytoma in case 2, and chromophobe RCC in case 3.

**Table 1:** Clinical and pathological features of the *mTOR*-mutated eosinophilic renal cell carcinomas of the present series

Case	Age	Gender	Size/laterality	Stage TNM	Surgery	Original diagnosis	Follow-up
1	21	F	9.5 cm/N.A	pT2aN1Mx	Radical nephrectomy	Unclassified RCC	30 months alive
2a*	58	F	6.5 cm/L	pT1bNxMx	Radical nephrectomy	Oncocytoma	108 months, alive with skull metastasis
2b*	58	F	3.1 cm/L	pT1aNxMx	Radical nephrectomy	Oncocytoma	108 months, alive with skull metastasis
2c*	69	F	Skull metastasis	pT1bNxM1	Metastasectomy	-	108 months, alive with skull metastasis
3a#	49	M	3.6 cm/R	pT1aNxMx	Partial nephrectomy	Chromophobe RCC	64 months, alive with liver metastases
3b#	52	M	Liver metastases	pT1aNxM1	Metastasectomy	-	64 months, alive with liver metastases
	(MD 35, SD ± 19.29)		(MD 6.5 cm, SD ± 4.17 cm)				

^*^Same patient (2a and 2b: renal tumors, 2c: skull metastasis)

^#^Same patient (3a: renal tumor, 3b: liver metastasis)

*F*, female; *M*, male; *L*, left; *R*, right; *N.A*., not available; *RCC*, renal cell carcinoma; *MD*, median; *SD*, standard deviation

**Fig. 1:**
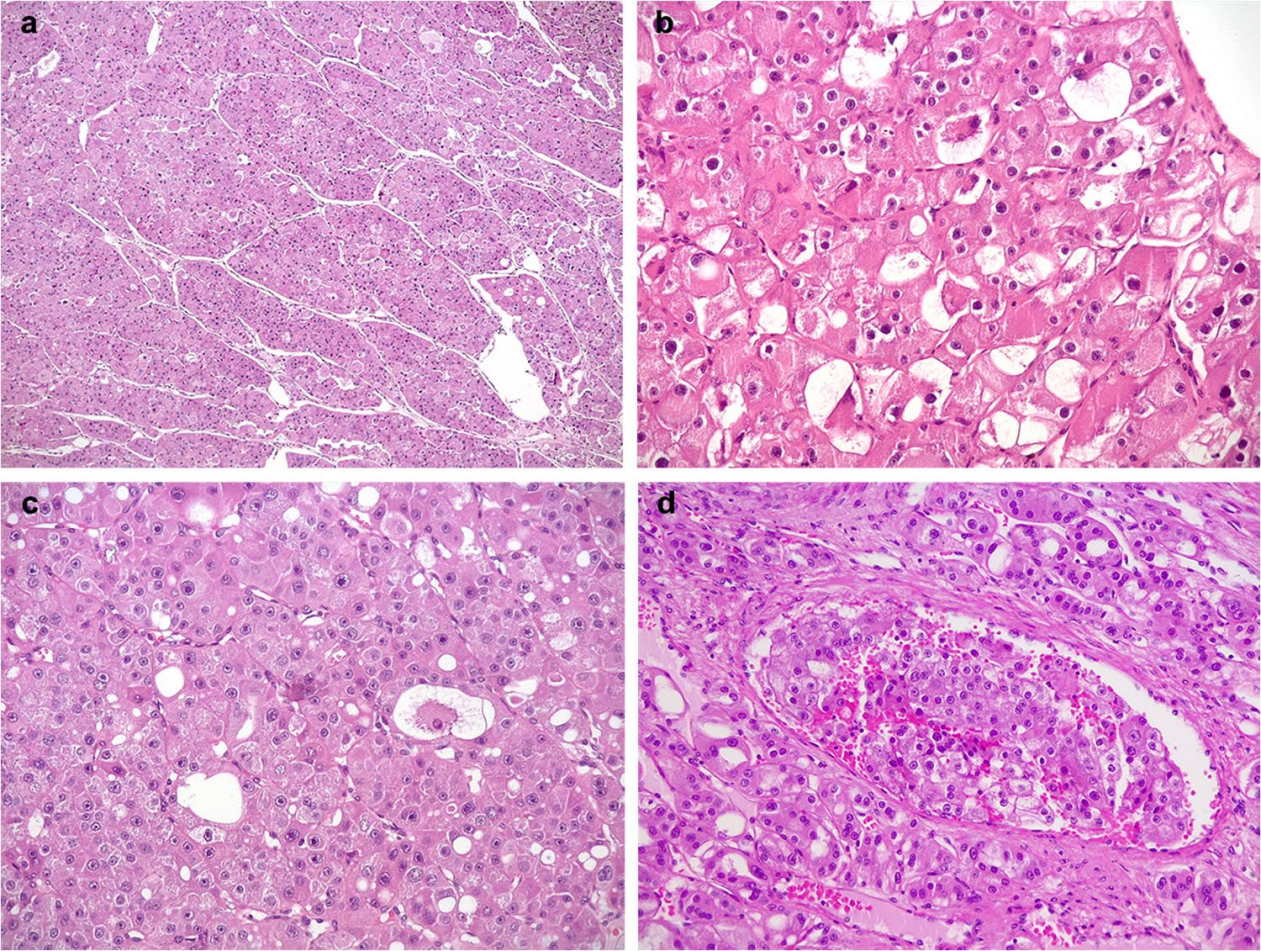
*mTOR-*mutated eosinophilic RCC. Low magnification demonstrates solid-nested growth (**a**). Large cells with perinuclear cytoplasmic shrinkage reminiscent of the so-called spider cells of cardiac rhabdomyomas were easily found in case 1 (**b**), or scattered in case 2 (**c**). Neoplastic vascular invasion was encountered in case 3 (**d**) (original magnification × 50 **a**, × 100 **b** and **c**, and × 200 **d**)

**Fig. 2:**
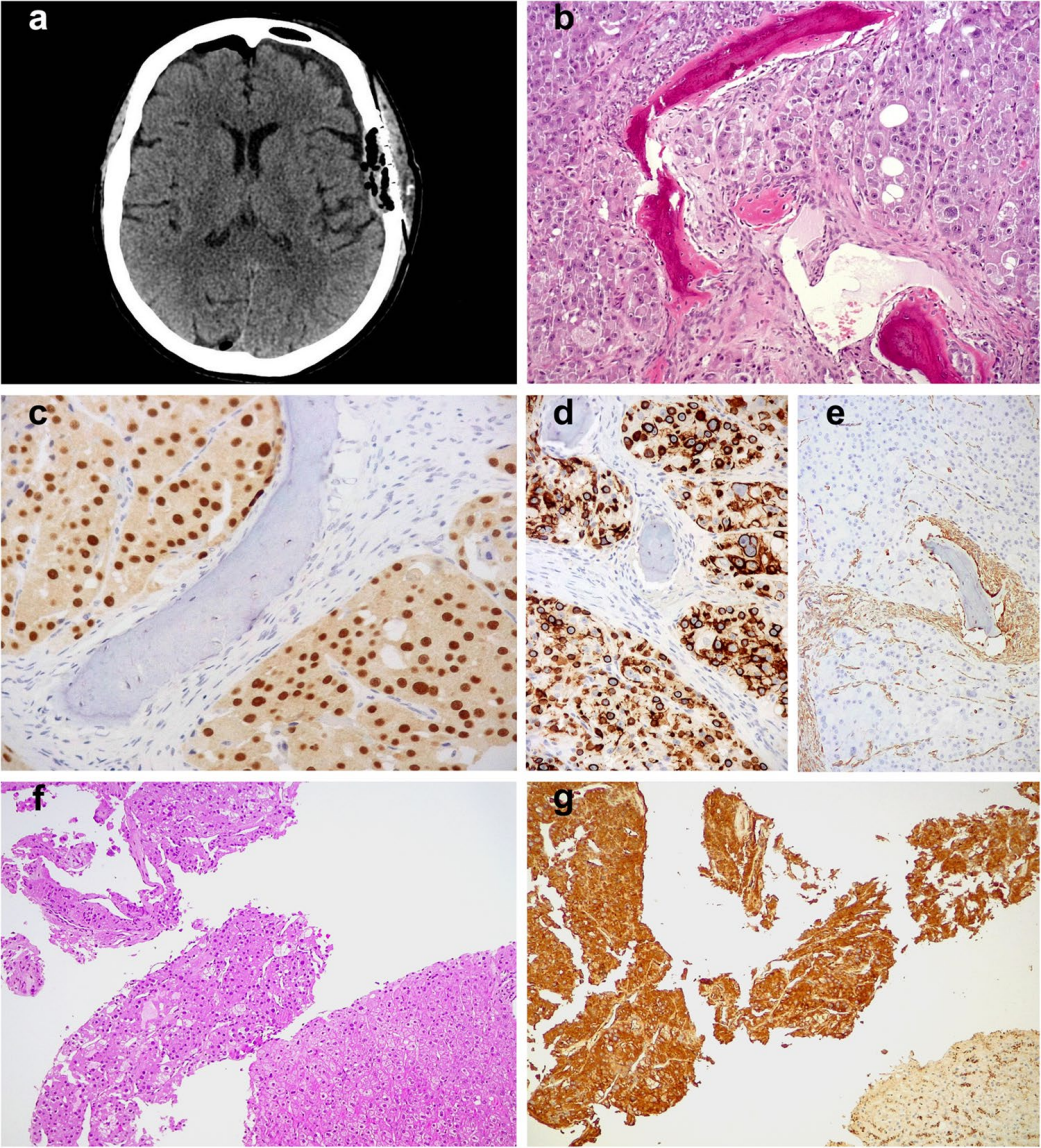
CT scan of case 2 revealed a skull mass (**a**) histologically characterized by large eosinophilic cells with round and enlarged nuclei (**b**). The neoplastic cells showed staining for PAX8 (**c**), cytokeratin 8–18 (**d**), but not for vimentin (**e**). The liver metastases of case 3 were histologically documented by biopsy (**f**) and were immunohistochemically positive for cathepsin-K (clone 3F9) (**g**) (original magnification × 50 **f** and **g**, × 100 **b**, **d**, and **e**, and × 200 **c**)

At light microscopy (Figs. 1 and 2), all the tumors were unencapsulated and composed of granular eosinophilic cells with cytoplasmatic vacuolization and nuclear atypia with prominent nucleoli (G3 by ISUP/WHO), mainly showing a solid/nested growth pattern. Additionally, in variable amount, there were larger cells with perinuclear cytoplasmic shrinkage and sparse basophilic Nissl-like granules, resembling the so-called spider cells of cardiac rhabdomyomas. Neoplastic cells were embedded within a fibrous and sometimes densely hyalinized stroma. Several thick-walled vessels were dispersed within the lesion along with peripherally entrapped normal renal tubules. Neither coagulative granular necrosis, mitoses, nor foamy macrophages were observed. In case 3, a neoplastic peritumoral vascular embolus was identified.

**Fig. 3:**
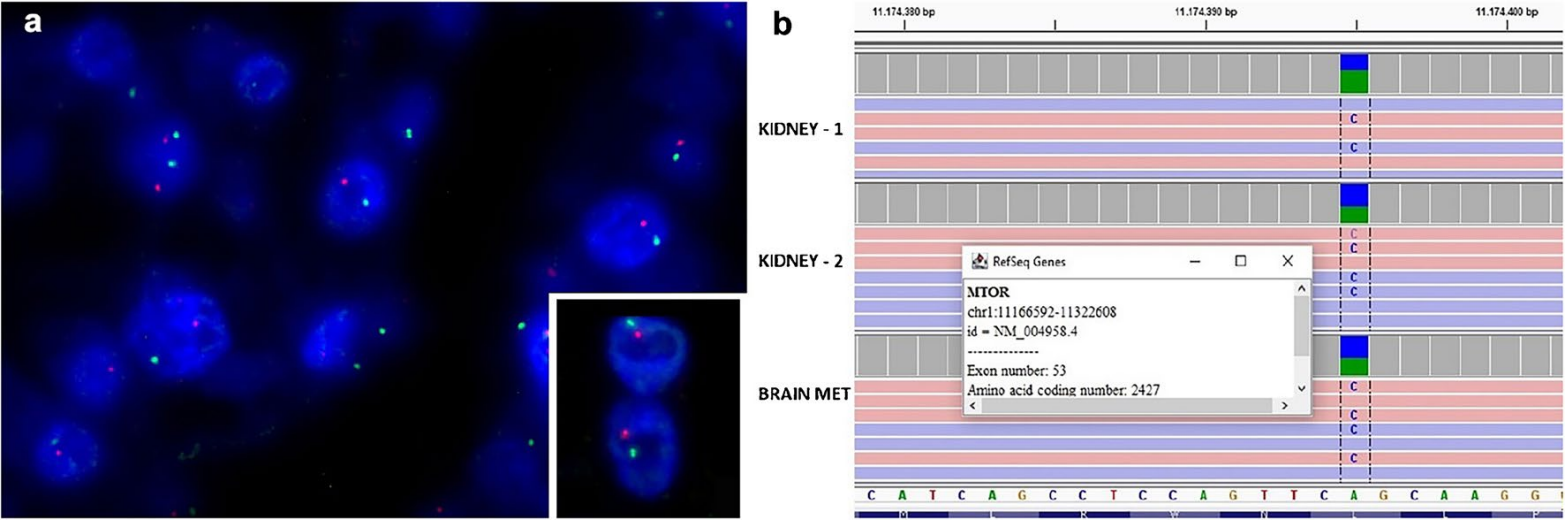
Loss of chromosome 1 identified by FISH in tumors with *mTOR* gene mutation (**a**). Pathogenic *mTOR* alterations in both primary renal tumors and the skull metastasis involving the exon 53 (p.Leu2427Arg) (**b**)

All the cases tested (Supplementary Table 2), expressed PAX8 along with immunolabelling for cathepsin-K (clone 3F9), whereas vimentin, and melanogenesis markers (HMB45 and Melan-A) were negative. Among the cytokeratins, strong and diffuse cytokeratin 8–18 expression was observed in all the neoplasms while in none of them significant staining of cytokeratin AE1/AE3 and cytokeratin 7 was found. All the cases were focally positive for CD117, expressed P70S6 Kinase and ph4E-BP1, and retained SDHB and FH. At FISH analysis all the cases showed 1p36/1q25 deletion (Fig. 3a); neither TFE3 nor TFEB rearrangements were identified.

One patient (case 1) had a synchronous small 9 mm nodal metastasis detected in one of seven regional lymph nodes and, to date, is alive without evidence of disease after 30 months. In another patient (case 2), a lesion was removed from the skull eleven years after surgical excision of the primary renal neoplasm. The skull lesion revealed overlapping histological and immunohistochemical findings with the kidney neoplasms and was therefore considered a metastasis. Finally, 3 years after surgery, two liver nodules were identified by imaging in the remaining patient (case 3); the largest of 3 cm as greater diameter was removed, whereas the other one was investigated by biopsy. Again, both liver lesions revealed overlapping histological and immunohistochemical findings with the renal tumor and were therefore considered metastases.

### Next-generation sequencing results

Genetic alterations in the *TSC* or *mTOR* pathway were found in all the samples tested with next-generation sequencing (Table 2).

**Table 2:** Molecular features of the *mTOR* mutated eosinophilic renal cell carcinomas of the present series

Case	Mutated gene	Exon	HGVS mutation	Protein mutation	Coverage	Quality	Frequency	Chromosomal position	Class
1	mTOR	30	c.4348_4368del	p.Tyr1450_Trp1456del	1200	6800	50%	chr1:11,217,309	Likely pathogenetic
2a*	mTOR	53	c.7280 T > G	p.Leu2427Arg	1200	11000	40%	chr1:11,174,395	Pathogenetic
2b*	mTOR	53	c.7280 T > G	p.Leu2427Arg	1200	11000	57%	chr1:11,174,395	Pathogenetic
2c*	mTOR	53	c.7280 T > G	p.Leu2427Arg	1300	8000	55%	chr1:11,174,395	Pathogenetic
3a#	mTOR	53	c.7280 T > A	p.Leu2427Gln	800	7000	66%	chr1:11,174,395	Pathogenetic
3b#	mTOR	53	c.7280 T > A	p.Leu2427Gln	1200	5700	45%	chr1:11,174,396	Pathogenetic

^*^Same patient (2a and 2b: renal tumors, 2c: skull metastasis)

^#^Same patient (3a: renal tumor, 3b: liver metastasis)

In case 1, a deletion in exon 30 of the *mTOR* gene (p.Tyr1450_Trp1456) was identified. This variant is reported both in COSMIC database (ref. COSM6972065) and OncoKB and occurs in the MTOR focal adhesion kinase targeting domain (FAT) domain, a key structural domain for the correct conformation of the catalytic pouch of the MTOR protein [26]. While its biological significance is still unknown, it has been identified as a statistically significant hotspot and is likely to be oncogenic. Moreover, it is also predicted to be pathogenic by the most common in-silico structural predictors (SIFT, POLYPHEN).

Case 2 showed pathogenic *mTOR* alterations in both primary renal tumors and the skull metastasis involving the exon 53 (p.Leu2427Arg—COSM2119114) (Fig. 3b).

Finally, case 3 showed pathogenic *mTOR* alterations in both the primary renal tumor and the liver metastases involving the exon 53 (p.Leu2427Gln—COSM1185313).

Both case 2 and case 3 variants involve the same aminoacidic residue of the MTOR Kinase Domain and are well known to be likely oncogenic [27].

No other pathogenetic mutation nor variant of unknown significance was identified in any of the primary tumor nor metastatic lesion. Furthermore, neither *mTOR* alterations nor *TSC1/TSC2* gene mutation were found in the normal renal parenchyma.

### Response to therapy

The patient of case 3 underwent radioembolization of the liver lesions. A new CT scan performed three months later documented the appearance of novel liver lesions (one lesion in the seventh hepatic segment 33 mm in long axis; 5 lesions in the fifth and sixth hepatic segments ranging from 17 to 47 mm in long axis; 1 lesion in the fourth hepatic segment 32 mm in long axis) and a pathological retrocaval lymphadenopathy (31 mm in short axis). Therefore, the patient started first-line systemic therapy with Pembrolizumab (200 mg intravenously, every 21 days) and Axitinib (5 mg orally twice a day). The best response achieved to this treatment was stable disease according RECIST 1.1, as shown by CT scans performed after 4, 8, and 12 months respectively. In the last examination, an osteolytic spot (22 mm in long axis) was identified at L2 so that the patient underwent external beam radiotherapy at this site (total dose: 20 Gy in 5 fractions). Fifteen months since the beginning of the systemic therapy, a new CT scan revealed further disease progression, with an increase in the size of all liver lesions, along with a new lesion in the eighth hepatic segment (6 mm in long axis) and new osteosclerotic spots in L5 and pelvic bone. Thus, shortly after first-line systemic treatment was permanently discontinued.

**Fig. 4:**
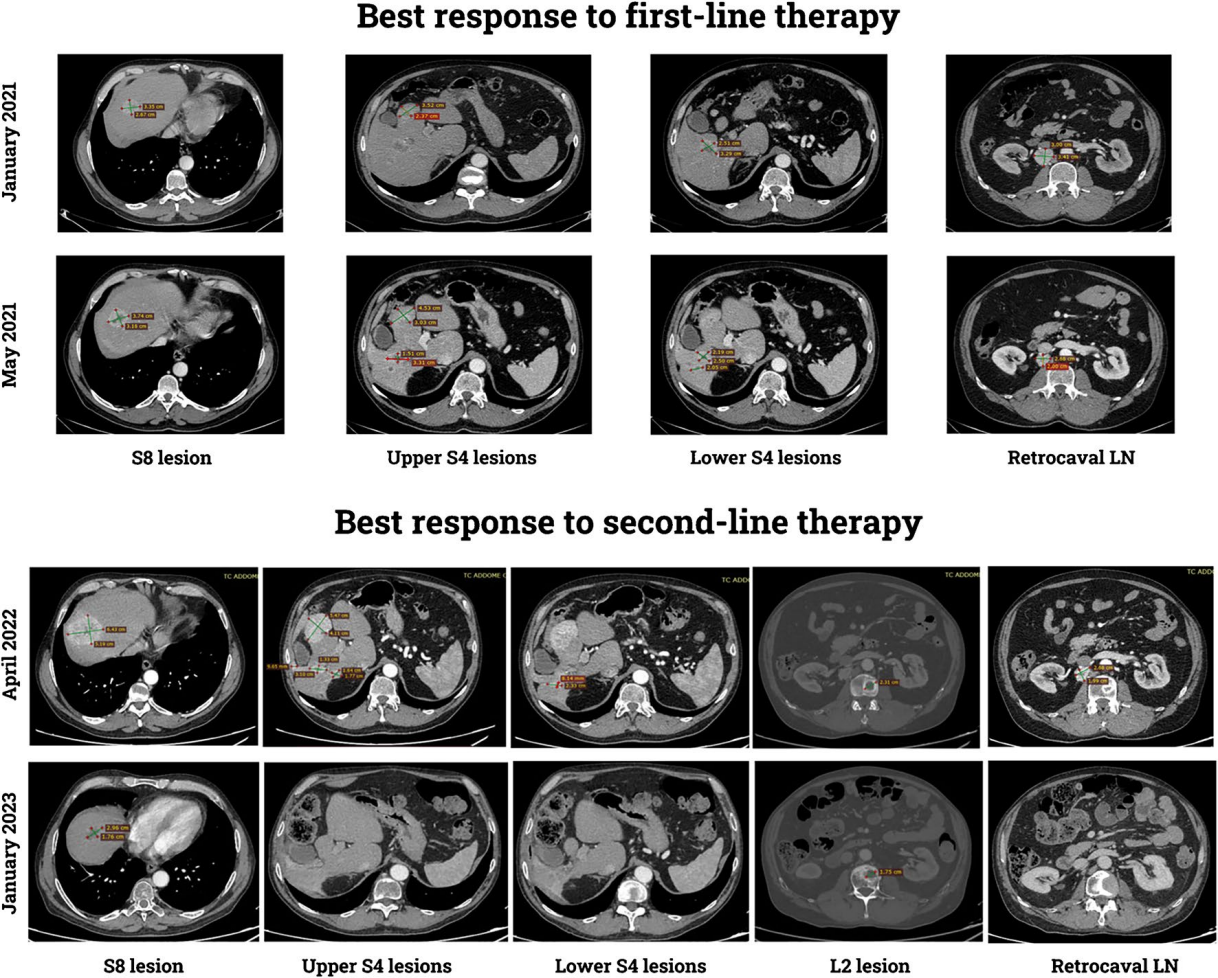
Comparison between the baseline CT scan performed before the start of the first-line therapy with Pembrolizumab and Axitinib (January 2021), at the best response to the first-line therapy (May 2021), at the progression to the first-line therapy (April 2022), and after 9 months of the second-line therapy with Lenvatinib and Everolimus. Little to any tumor shrinkage was observed with Pembrolizumab and Axitinib. Conversely, following Lenvatinib and Everolimus, just a few hepatic lesions were detected, showing a decrease in size and vascularization. Disease stability for the osteolytic lesion in L2 was also recorded. All the images were taken in the arterial phase

Based on the molecularly documented *mTOR* mutation (L2427Q, exon 53), the choice for second-line therapy was towards a combination of Lenvatinib (18 mg daily) and Everolimus (5 mg daily) in an off-label regimen. Within 4 months several treatment-related adverse events were observed including G2 hypertension, G2 fatigue, G2 diarrhea, and G2 mucositis. Hence, the dose of Lenvatinib was initially reduced to 14 mg daily and then to 10 mg daily. The first follow-up CT scan, after two months, showed reduction in size and vascularization of both all the target liver lesions and the retrocaval lymphadenopathy. Four months later, another CT scan revealed a further dimensional reduction of the liver lesions. Finally, 8 months after the beginning of the second-line therapy, the last CT scan available documented a numerical decrease in the liver lesions (with only two of them detectable to date) and an additional shrinkage of the retrocaval lymphadenopathy (< 10 mm in short axis) (Fig. 4). Currently, the patient is still on Lenvatinib (10 mg orally once a day) and Everolimus (5 mg orally once a day) treatment with fair tolerance.

## Discussion

In this study, we reported four sporadic high-grade eosinophilic RCCs occurring in three patients with histologically documented metastases, characterized by the presence of “spider cells,” the immunohistochemical expression of cathepsin-K (clone 3F9), and mutations of *mTOR* gene.

In the last years, the differential diagnosis of eosinophilic tumors has become even more challenging for pathologists due to growing wide spectrum of tumors with oncocytic cells, usually characterized by non-aggressive behavior (Table 3) [1]. In this morphological scenario, cathepsin-K (clone 3F9) is a useful tool for differentiating renal oncocytoma, chromophobe RCC, and LOT which are negative for cathepsin-K (clone 3F9), from ESC-RCC, EVT, and mTOR-mutated eosinophilic RCC which are positive for this marker [28]. Whether the recognition of ESC-RCC is usually straightforward for uropathologists, EVT and mTOR-mutated eosinophilic RCC may show overlapping features, as highlighted by Tjota et al. [22]. However, EVT has prominent or extreme cytoplasmic vacuolation whereas in mTOR-mutated eosinophilic RCCs, we observed large cells with perinuclear cytoplasmic shrinkage resembling the so-called spider cells of cardiac rhabdomyomas occurring, interestingly, in tuberous sclerosis patients. This finding is morphologically reliable since similar elements are also focally reported by Tjota et al. as “somewhat rhabdoid appearance” [22].

**Table 3:** Key morphological and immunohistochemical hallmarks of eosinophilic renal neoplasms

Tumor	Key histological features	PAX 8	CAT. K	PAN-CKs	CK 7	CD 117	CK 20	Vimentin	GATA 3	PV	S100 A1	AMACR	CA 9	SDH-B	HMB 45	Melan-A	ALK
Chromophobe RCC, eosinophilic	Solid (“plant-like”) architecture, prominent cell membranes, raisinoid nuclei with perinuclear halos	+	Neg	+	+	+	Neg	Neg	Neg	+	Neg	Neg	Neg	Retained	Neg	Neg	Neg
Renal oncocytoma	Solid-nested to tubulocystic growth, central edematous-scary areas, no perinuclear halos	+	Neg	+	Neg	+	Neg	Neg	Neg	+	+	Neg	Neg	Retained	Neg	Neg	Neg
Hybrid oncocytic tumor	Admixed chromophobe and oncocytoma-like features (i.e. solid-nested architecture but with perinuclear halos)	+	N.A	+	±	+	Neg	Neg	N.A	±	-/ +	Neg	Neg	Retained	Neg	Neg	Neg
Clear cell RCC, eosinophilic	Delicate capillary vessels network, usually at least scattered conventional clear cell areas	+	Neg	+	Neg	Neg	Neg	+	Neg	Neg	+	-/ +	+	Retained	Neg	Neg	Neg
Papillary RCC, oncocytic	Papillary-tubular architecture (at least focally)	+	Neg	+	+	Neg	Neg	+	Neg	Neg	+	+	Neg	Retained	Neg	Neg	Neg
LOT	Alternating solid-trabecular cellular areas with loose edematous stromal zones and fresh hemorrhage	+	Neg	+	+	Neg	Neg	Neg	+	N.A	+	Neg	Neg	Retained	Neg	Neg	Neg
ESC-RCC	Solid and cystic architecture, sparse foamy macrophages	+	+	+	Neg	Neg	+	+	Neg	Neg	+	Neg	Neg	Retained	Neg	Neg	Neg
*SDH*-deficient RCC	Solid-nested architecture, low-grade cells often with flocculent cytoplasm inclusions	+	Neg	Neg	Neg	Neg	Neg	Neg	N.A	N.A	N.A	Neg	Neg	Absent	Neg	Neg	Neg
Epithelioid angiomyolipoma	Epithelioid sometimes pleomorphic cells	Neg	+	Neg	Neg	Neg	Neg	Neg	N.A	Neg	Neg	Neg	Neg	Retained	+	+	Neg
*ALK-*rearranged RCC	Large cells displaying various architectural growth patterns, focally mucin deposit	+	Neg	+	+	Neg	Neg	+	Neg	N.A	N.A	+	Neg	Retained	Neg	Neg	+
*mTOR*-mutated eosinophilic RCC	Cytoplasmic vacuolation and shrinkage with basophilic Nissl-like granules “spider cells,” nuclear atypia with prominent nucleoli	+	+	+	Neg	-/ +	Neg	Neg	Neg	Neg	-/ +	-/ +	Neg	Retained	Neg	Neg	Neg

Abbreviations: *RCC*, renal cell carcinoma; *CAT*. *K*, cathepsin K; *CK*, cytokeratin; *PV*, parvalbumin; *AMACR*, alpha-methylacyl-CoA racemase; *CA9*, carbonic anhydrase IX; *SDH*, succinate dehydrogenase; *ALK*, anaplastic lymphoma kinase; *N.A*., not available; *LOT*, low-grade oncocytic tumor; *ESC*, eosinophilic, solid, and cystic

Activating *mTOR* gene mutations are the genetic hallmarks of these high-grade eosinophilic RCCs. In accordance with the activation of the *mTOR* pathway, we observed an overexpression of two proteins downstream of the *mTOR* pathway, namely, the phosphorylated forms of 4EBP1 and S6K. Moreover, loss of chromosome 1 was identified in the four renal neoplasms and the skull and liver metastases, which represents the genomic location of the *mTOR* gene. The association of loss of chromosome 1 along with activating *mTOR* mutations has been also reported by Tjota et al. [22]. Being mTORC1 a dimer, it is possible to speculate that a heterodimer of wild-type and mTOR mutant proteins may not confer sufficient mTOR activation [29].

Recently, several renal tumors harboring *mTOR* gene mutations have been described (Table 4). In our comprehensive analysis of 57 neoplasms reported in the literature and our series, *mTOR* gene mutations have been observed in 16 EVTs, 24 LOTs, and 17 RCCs. The percentage of *mTOR* gene mutations in the molecularly tested cases is 31% of EVT (16 of 51 tumors) and 30.7% of LOT (24 of 78 tumors). Interestingly, the same *mTOR* gene mutation (L2427) has been detected in 11 of 16 (68.7%) EVTs, in 9 of 24 (37.5%) LOTs, and in 9 of 17 (53%) mTOR-mutated RCCs. The high prevalence of this hotspot genetic alteration, which falls in the catalytic subunit of the mTOR protein, may confer a selective advantage. Moreover, it has been demonstrated both in vitro and in vivo that cells harboring this mutation are highly sensitive to mTOR inhibitors [27, 30].

**Table 4:** *mTOR*-mutated eosinophilic renal cell carcinomas

Case	Reference	Gender	Age	Size/laterality	Diagnosis	mTOR mutation	chr 1 loss	Follow-up
1	He et al., 2018, Farcas et al., 2021	M	54	2.6 cm/N.A	EVT	mTOR, c.5930C > A	loss	50 months, NED
2	Chen et al., 2019	F	68	4.4 cm/N.A	EVT	mTOR, p.Leu2427Arg	loss	13 months, NED
3	Chen et al., 2019	M	59	3.6 cm/N.A	EVT	mTOR, p.Leu2427Arg	loss	10 months, NED
4	Kapur et al. 2021	M	55	1.8 cm/L	EVT	mTOR, p.Leu2427Gln	loss	15 months, NED
5	Farcas et al. 2021	F	31	3.5 cm/N.A	EVT	mTOR, c.7280 T > G	*	31 months, NED
6	Farcas et al. 2021	M	25	3.8 cm/N.A	EVT	mTOR, c.7257_7259delinsTGT	*	75 months, NED
7	Farcas et al. 2021	M	72	3.5 cm/N.A	EVT	mTOR, c.7280 T > A	*	144 months, NED
8	Farcas et al. 2021	F	59	4 cm/N.A	EVT	mTOR, c.7280 T > C	*	18 months, NED
9	Farcas et al. 2021	M	15	11.5 cm/N.A	EVT	mTOR, c.7280 T > G	*	19 months, NED
10	Farcas et al. 2021	F	69	4 cm/N.A	EVT	mTOR, c.4343_4363del	*	47 months, NED
11	Farcas et al. 2021	M	42	7 cm/N.A	EVT	mTOR, c.7280 T > A	*	18 months, NED
12	Xia et al. 2022	M	42	3.5 cm/N.A	EVT	mTOR c.7280 T > A	N.A	N.A
13	Xia et al. 2022	F	32	2.5 cm/N.A	EVT	mTOR c.7280 T > G	N.A	N.A
14	Xia et al. 2022	M	24	6 cm/N.A	EVT	mTOR c.7280 T > G	N.A	N.A
15	Xia et al. 2022	M	59	2.5 cm/N.A	EVT	mTOR c.7237_7238delinsCT	N.A	N.A
16	Xia et al. 2022	M	47	2.5 cm/N.A	EVT	mTOR c.11C > T + TSC2 c.3352C > T	N.A	N.A
1	Tjota et al., 2020	F	66	4.1 cm/L	LOT	mTOR c.7280 T > A	N.A	6 months NED
2	Tjota et al., 2020	M	66	2.5 cm/R	LOT	mTOR c.5930C > G	no loss	156 months NED
3	Morini et al., 2021	F	57	3.7 cm/L	LOT	mTOR c.6644 C > T	N.A	7 months NED
4	Morini et al., 2021	F	61	3.8 cm/R	LOT	mTOR c.7499 T > A	N.A	N.A
5	Morini et al., 2021	F	78	3.7 cm/R	LOT	mTOR c.6644 C > A	N.A	N.A
6	Morini et al., 2021	F	83	3.5 cm/R	LOT	mTOR c.4348 T > G	N.A	49 died of other disease
7	Morini et al., 2021	F	79	8.5 cm/R	LOT	mTOR c.320_323delinsATTT	N.A	49 months NED
8	Morini et al., 2021	F	58	5.5 cm/R	LOT	mTOR c.7280 T > A	N.A	N.A
9	Morini et al., 2021	F	76	3.7 cm/R	LOT	mTOR c.7498 A > T	N.A	N.A
10	Kapur et al., 2021	F	79	7.8 cm/R	LOT	mTOR p.Leu2427Gln	no loss	26 months died
11	Kapur et al., 2021	F	86	6.5 cm/R	LOT	mTOR p.Ser2215Tyr	no loss	3 months NED
12	Kapur et al., 2021	F	71	3.8 cm/R	LOT	mTOR p.Ser2413Leu	no loss	53 months NED
13	Kapur et al., 2021	F	75	2.4 cm/L	LOT	mTOR p.Lys1452_Glu1455del	no loss	36 months died
14	Zhang et al., 2022	F	79	1.6 cm/R	LOT	mTOR p.Leu2427Gln + TSC2 p.Met286Val	N.A	33 months NED
15	Mohanty et al., 2021	F	65	2.3 cm/L	LOT	mTOR c.7280G > A	N.A	2 months NED
16	Williamson et al., 2023	F	61	8 cm/N.A	LOT	mTOR c.7500 T > G	N.A	N.A
17	Williamson et al., 2023	F	73	6.9 cm/N.A	LOT	mTOR c.7280 T > A	N.A	N.A
18	Williamson et al., 2023, Trpkov 2019	F	63	5.2 cm/N.A	LOT	mTOR c.6644C > T + TSC1 c.2356C > T	N.A	N.A
19	Williamson et al., 2023, Trpkov 2019	F	61	3 cm/N.A	LOT	NF2 mTOR c.4448G > T	N.A	N.A
20	Williamson et al., 2023	F	41	2 cm/N.A	LOT	mTOR c.7498A > T	N.A	N.A
21	Chen et al., 2023	F	45	2.5 cm/R	LOT	mTOR p.L2427Q	N.A	57 months NED
22	Chen et al., 2023	M	65	3 cm/R	LOT	mTOR p.L2427Q	N.A	53 months NED
23	Chen et al., 2023	F	66	3.5 cm/R	LOT	mTOR p.L2427Q	N.A	32 months NED
24	Chen et al., 2023	F	65	3 cm/R	LOT	mTOR p.L2427Q	N.A	10 months NED
1	Romero et al., 2020	F	64	N.A	Eosinophilic chromophobe	mTOR I2501F	N.A	5 months NED
2	Romero et al., 2020	F	50	N.A	Classic chromophobe	mTOR S2215F	N.A	160 months NED
3	Romero et al., 2020	M	63	N.A	Eosinophilic chromophobe	mTOR I2500F	N.A	41 months NED
4	Romero et al., 2020	F	58	N.A	Eosinophilic chromophobe	mTOR L2427R	N.A	13 months NED
5	Romero et al., 2020	F	62	N.A	Eosinophilic chromophobe	mTOR L2427R	N.A	10 months NED
6	Romero et al., 2020	F	44	N.A	Eosinophilic chromophobe	mTOR V2006F	N.A	46 months NED
7	Romero et al., 2020	F	75	N.A	N.A	mTOR L2427R	N.A	24 months metastasis
8	Romero et al., 2020	F	75	N.A	Eosinophilic chromophobe	mTOR E1613Q	N.A	24 months NED
9	Tjota et al., 2021	M	65	8.5 cm/N.A	Eosinophilic carcinoma	mTOR, c.7279_7280delinsAA, p.Leu2427Lys	loss	Synchronous lymph node, liver and lung metastasis, N.A
10	Chen et al., 2016	N.A	N.A	N.A	Unclassified RCC	mTOR L2427R	N.A	N.A
11	Chen et al., 2016	N.A	N.A	N.A	Unclassified RCC	mTOR L2427R	N.A	N.A
12	Chen et al., 2016	N.A	N.A	N.A	Unclassified RCC	mTOR L2427R	N.A	N.A
13	Chen et al., 2016	N.A	N.A	N.A	Unclassified RCC	mTOR I1973F	N.A	N.A
14	Chen et al., 2016	N.A	N.A	N.A	Unclassified RCC	mTOR V2475M	N.A	N.A
15	Present study	F	21	9.5 cm/L	Eosinophilic carcinoma	mTOR, c.4348_4368del p.Tyr1450_Trp1456del	loss	Lymph node metastasis, 30 months alive
16	Present study	F	58	6.5 cm/L	Eosinophilic carcinoma	mTOR, c.7280 T > G, p.Leu2427Arg	loss	Skull metastasis after 132 months, 135 months alive
17	Present study	M	49	3.6 cm/L	Eosinophilic carcinoma	mTOR, c.7280 T > A,p.Leu2427Gln	loss	Liver metastasis after 36 months, 64 months alive

^*^Not revealed but using other less specific technique

Abbreviations: *chr*, chromosome; *M*, male; *F*, female; *R*, right; *L*, left; *N.A*. not available; *EVT*, eosinophilic vacuolated tumor; *LOT*, low-grade oncocytic tumor; *NED*, no evidence of disease

Differently from other eosinophilic tumors harboring *mTOR* mutations, the three patients presented in this study developed metastases. In case 1, the site of metastasis was a small lymph node of the renal sinus (i.e., still a loco-regional disease), which was removed together with the primary kidney lesion; in case 2, the metastasis was larger and occurred in the skull (i.e., a frankly metastatic disease) eleven years later the first diagnosis; finally, in case 3, multiple metastases in the liver were observed after 3 years from nephron-sparing surgery. Because of the amount of tumor available, we were able to test also the liver and skull metastases by immunohistochemistry and genetic analysis. We found morphological and immunohistochemical features (PAX8, cytokeratin 8–18, cathepsin-K clone 3F9 positivity, and vimentin negativity) overlapping with those observed in the primary renal tumors. The same *mTOR* gene mutation was identified in the liver metastasis and the primary renal tumor, as well as in the skull metastasis and the two renal tumors. A possible explanation of the latter finding is that one mass represented the intrarenal metastasis of the other tumor. Despite this possibility being well-known in other RCCs, it seems unlikely in this case since the skull metastasis occurred 11 years after the radical nephrectomy, suggesting instead a slow progression of the disease. Recently, Tjota et al. reported a similar case with liver metastasis harboring *mTOR* gene mutation (p.L2427K) and loss of chromosome 1 [22]. To date, the predictive value of *mTOR* gene mutations in metastatic RCC patients treated with mTOR inhibitors remains controversial, with studies suggesting such a predictive role [31], and others not [32]. A multicenter, histology-agnostic, single-arm prospective phase II trial of the mTORC1 inhibitor, Everolimus, in patients with solid tumors mainly harboring *TSC1/TSC2* mutations ultimately failed, showing no association between these genomic alterations and response to targeted treatment in a broad spectrum of neoplasms, not including RCCs [33]. In our study, the clinical response to mTOR inhibitors observed in patient 3 might support the hypothesis that *mTOR* alterations could predict response to mTOR inhibitors, although our patient received a combination of one mTOR inhibitor, plus a multikinase inhibitor (mainly targeting VEGFRs), making it impossible to dissect the relative contribution of the two different classes of agents to the observed activity and efficacy.

In conclusion, herein, we present a distinct renal tumor characterized by high-grade eosinophilic cells, cathepsin-K (clone 3F9) immunohistochemical expression, and harboring *mTOR* gene mutation demonstrating a malignant potential and showing responsiveness to an mTOR inhibitor-containing combination. This latter observation encourages pathologists to investigate *mTOR* gene mutation in aggressive high-grade/cathepsin-K-positive eosinophilic RCC.

### Supplementary Information

Below is the link to the electronic supplementary material.

Supplementary file1 (DOCX 17 KB)

Supplementary file2 (DOCX 18 KB)

## Data Availability

All data generated or analysed during this study are included in this published article [and its supplementary information files].
