# Supplementary material for: mTOR eosinophilic renal cell carcinoma: a distinctive tumor characterized by mTOR mutation, loss of chromosome 1, cathepsin-K expression, and response to target therapy
Source: Virchows Arch. 2023 Nov 8;483(6):821–33. doi: 10.1007/s00428-023-03688-2 (PMC10700445; doi:10.1007/s00428-023-03688-2)
Supplement: Supplementary file 1 — Supplementary file1 (DOCX 17 KB) [file 428_2023_3688_MOESM1_ESM.docx]

**Table S1.** List of antibodies used in the present series.

| **Antibody** | **Clone** | **Provider** | **Dilution** |
| --- | --- | --- | --- |
| PAX 8 | MR1-50 | ROCHE | Prediluted |
| CAT. K | 3F9 | ABCAM | 1:1600 |
| HMB 45 | HMB45 | ROCHE | Prediluted |
| Melan-A | A103 | ROCHE | Prediluted |
| CK 8-18 | 5D3, LEICA | LEICA | 1:100 |
| CK 7 | OV-TL 12/30 | LEICA | 1:100 |
| CK 20 | PW31 | LEICA | 1:100 |
| CK AE1/AE3 | AE1&AE3 | LEICA | 1:100 |
| CD 117 | T595 | LEICA | 1:10 |
| PV | 2E11 | ABCAM | 1:500 |
| S100 A1 | EPR5250 | ABCAM | 1:100 |
| GATA 3 | L50-823 | BD PHARMIGEN | 1:150 |
| Vimentin | V9 | LEICA | 1:400 |
| CA 9 | TH22 | LEICA | 1:100 |
| CD 10 | 56C6 | LEICA | 1:50 |
| CD 13 | 38C12 | LEICA | 1:100 |
| AMACR | EPMU1 | LEICA | 1:200 |
| FH | J-13 | DIAPATH | 1:50 |
| SDH | EPR10880 | ABCAM | 1:1000 |
| MUC 1 | 695 | BIOCARE | Prediluted |
| EMA | GP1.4 | LEICA | 1:100 |
| Cyclin D1 | EP12 | LEICA | Prediluted |
| Ki67 | K2 | LEICA | Prediluted |
| TFE 3 | MRQ37 | MONOSAN | Prediluted |
| TFE B | POLYCLONAL | BETHYL | 1:1000 |
| P70S6 Kinase | 49D7 | CELL SIGNALING | 1:1000 |
| ph4E-BP1 | 236B4 | CELL SIGNALING | 1:400 |

Abbreviations: CAT. K: cathepsin K, CK: cytokeratin, PV: parvalbumin, CA 9: carbonic anhydrase IX, AMACR: alpha-methylacyl-CoA racemase, FH: fumarate hydratase, SDH: succinate dehydrogenase.
