# Supplementary material for: mTOR eosinophilic renal cell carcinoma: a distinctive tumor characterized by mTOR mutation, loss of chromosome 1, cathepsin-K expression, and response to target therapy
Source: Virchows Arch. 2023 Nov 8;483(6):821–33. doi: 10.1007/s00428-023-03688-2 (PMC10700445; doi:10.1007/s00428-023-03688-2)
Supplement: Supplementary file 2 — Supplementary file2 (DOCX 18 KB) [file 428_2023_3688_MOESM2_ESM.docx]

**Table S2.** Immunohistochemical and FISH findings of the *mTOR* mutated eosinophilic renal cell carcinomas of the present series.

| **Case** | **PAX 8** | **CAT. K** | **HMB 45** | **Melan-A** | **CK 8-18** | **CK 7** | **CK 20** | **CK AE1/AE3** | **CD 117** | **PV** | **S100 A1** | **GATA 3** | **Vimentin** | **CA 9** | **CD 10** | **CD 13** | **AMACR** | **FH** | **SDH** | **MUC 1** | **EMA** | **Cyclin D1** | **Ki67** | **TFE 3** | **TFEB** | **P70S6** | **ph4E-BP1** | **FISH TFE3/TFEB** |
| --- | --- | --- | --- | --- | --- | --- | --- | --- | --- | --- | --- | --- | --- | --- | --- | --- | --- | --- | --- | --- | --- | --- | --- | --- | --- | --- | --- | --- |
| 1 | 10% + | 90% + | Neg. | Neg. | 90% + | 1% + | 5% + | 5% + | 10% + | 60% + | 60% + | Neg. | Neg. | Neg. | 10% + | Neg. | 20% + | Retained | Retained | <1% + | <1% + | 10% + | 2% + | Neg. | Neg. | 90% + | 80% + | Neg. |
| 2a* | 10% + | 90% + | Neg. | Neg. | 60% + | Neg. | Neg. | Neg. | 10% + | Neg. | 70% + | Neg. | Neg. | Neg. | 15% + | Neg. | Neg. | Retained | Retained | 20% + | 10% + | Neg. | 1% + | Neg. | Neg. | 80% + | 90% + | Neg. |
| 2b* | 80% + | 90% + | Neg. | Neg. | 90% + | Neg. | Neg. | 1% + | 10% + | Neg. | 40% + | Neg. | Neg. | Neg. | 20% + | Neg. | 30% + | Retained | Retained | < 1% + | < 1% + | 15%+ | 2%+ | Neg. | Neg. | 90% + | 90% + | Neg. |
| 2c* | 90% + | 20% + | Neg. | Neg. | 90% + | Neg. | 40% + | Neg. | 20% + | 15% + | 90% + | Neg. | Neg. | Neg. | 80% + | Neg. | 30% + | Retained | Retained | 5% + | 10% + | 10% + | 5%+ | Neg. | Neg. | 70% + | 80% + | Neg. |
| 3a# | 70% + | 80% + | Neg. | Neg. | 90% + | Neg. | Neg. | 5% + | 10% + | Neg. | 5% + | Neg. | Neg. | Neg. | 60% + | Neg. | Neg. | Retained | Retained | 30% + | 80% + | 10% + | 1% + | Neg. | Neg. | 90% + | 70% + | Neg. |
| 3b# | 10% + | 80% + | Neg. | Neg. | 10% + | Neg. | Neg. | 5% + | 10% + | Neg. | Neg. | Neg. | Neg. | Neg. | 80% + | Neg. | Neg. | Retained | Retained | 10% + | 90% + | 5% + | 10% + | Neg. | Neg. | 80% + | 90% + | Neg. |

*same patient (2a and 2b: renal tumors, 2c: skull metastasis)

#same patient (3a: renal tumor, 3b: liver metastasis)

Abbreviations: CAT. K: cathepsin K, CK: cytokeratin, PV: parvalbumin, CA 9: carbonic anhydrase IX, AMACR: alpha-methylacyl-CoA racemase, FH: fumarate hydratase, SDH: succinate dehydrogenase.
